# Conventional 3D conformal radiotherapy and volumetric modulated arc therapy for cervical cancer: Comparison of clinical results with special consideration of the influence of patient- and treatment-related parameters

**DOI:** 10.1007/s00066-021-01782-5

**Published:** 2021-05-03

**Authors:** Leif Hendrik Dröge, Franziska-Felicitas von Sivers, Markus Anton Schirmer, Hendrik Andreas Wolff

**Affiliations:** 1 Department of Radiotherapy and Radiation Oncology, University Medical Center Göttingen, Robert-Koch-Straße 40, 37075 Göttingen, Germany; 2 University Medical Center Göttingen, Göttingen, Germany; 3 Department of Radiology, Nuclear Medicine and Radiotherapy, Radiologie München, 80333 Munich, Germany; 4 Department of Radiotherapy and Radiation Oncology, University Medical Center Regensburg, Regensburg, Germany

**Keywords:** Gynecologic cancer, Radiochemotherapy, Intensity-modulated radiotherapy, Urinary toxicity, Small bowel toxicity, Body mass index

## Abstract

**Purpose:**

Intensity-modulated radiotherapy (IMRT) for cervical cancer yields favorable results in terms of oncological outcomes, acute toxicity, and late toxicity. Limited data are available on clinical results with volumetric modulated arc therapy (VMAT). This study’s purpose is to compare outcome and toxicity with VMAT to conventional 3D conformal radiotherapy (3DCRT), giving special consideration to the influence of patient- and treatment-related parameters on side effects.

**Materials and methods:**

Patients with cervical cancer stage I–IVA underwent radiotherapy alone or chemoradiotherapy using 3DCRT (*n* = 75) or VMAT (*n* = 30). Survival endpoints were overall survival, progression-free survival, and locoregional control. The National Cancer Institute Common Terminology Criteria for Adverse Events and the Late Effects of Normal Tissues criteria were used for toxicity assessment. Toxicity and patient- and treatment-related parameters were included in a multivariable model.

**Results:**

There were no differences in survival rates between treatment groups. VMAT significantly reduced late small bowel toxicity (OR = 0.10, *p* = 0.03). Additionally, VMAT was associated with an increased risk of acute urinary toxicity (OR = 2.94, *p* = 0.01). A low body mass index (BMI; OR = 2.46, *p* = 0.03) and overall acute toxicity ≥grade 2 (OR = 4.17, *p* < 0.01) were associated with increased overall late toxicity.

**Conclusion:**

We demonstrated significant reduction of late small bowel toxicity with VMAT treatment, an improvement in long-term morbidity is conceivable. VMAT-treated patients experienced acute urinary toxicity more frequently. Further analysis of patient- and treatment-related parameters indicates that the close monitoring of patients with low BMI and of patients who experienced relevant acute toxicity during follow-up care could improve late toxicity profiles.

**Supplementary Information:**

The online version of this article (10.1007/s00066-021-01782-5) contains supplementary material, which is available to authorized users.

## Introduction

Radiotherapy and chemoradiotherapy (RT/CRT) reduce local and distant recurrence and improve survival in cervical cancer [1, 2], not seldom at the expense of side effects [3–6]. Recently, intensity-modulated radiotherapy (IMRT) and volumetric modulated arc therapy (VMAT) were introduced into radiation oncology practice [7, 8]. IMRT was demonstrated to achieve favorable results in terms of oncological outcomes and toxicity [9–12]. VMAT, at the planning level, achieved excellent dose distributions [13, 14]. On a clinical level, a few studies have reported favorable toxicity profiles or promising outcomes with VMAT, whereby these studies focused on adjuvant treatment [15], neoadjuvant treatment [16], or treatment in elderly patients [17]. However, comparisons of VMAT with other external beam radiotherapy (EBRT) techniques are still rare [18].

We introduced VMAT to our clinic in 2009. The purpose of the current study was to compare clinical results of 3D conformal radiotherapy (3DCRT) and VMAT when treating cervical cancer. The endpoints were outcome and toxicity. We included patient- and treatment-related parameters with a possible influence on side effects in multivariable analysis.

## Patients and methods

### Patients

We included patients who were treated with RT/CRT for cervical cancer of Fédération Internationale de Gynécologie et d`Obstétrique (FIGO) stages I–IVA. Patients with distant metastases or paraaortic lymph node spread were excluded. The staging procedures were performed according to the respective guidelines [19, 20] at our gynecological cancer center or at a hospital selected by the patient. Patients received abdominal ultrasound and chest radiograph or a CT scan of the chest and abdomen. A pelvic MRI scan was performed for local tumor staging. A rectoscopy or cystoscopy was performed in patients with clinical or radiological suspicion of invasion into rectum or bladder. According to local practice, surgical staging was not routinely performed. The treatment strategies (e.g., definitive RT/CRT vs. primary surgery) were discussed and determined on an individual basis in the multidisciplinary tumor board. Owing to the changes in treatment strategies over the study period of approximately two decades and to the retrospective study design, it is difficult to further concretize and generalize the indications. Overall, patients with FIGO stages IIIA-IVA were preferably treated with definitive RT/CRT. The options in patients with FIGO stage IIB were, depending on further clinical factors, primary surgery, definitive RT/CRT, and neoadjuvant RT/CRT. Patients with FIGO stages I–IIA preferably underwent primary surgery. The indication for adjuvant RT/CRT was determined depending on histopathological adverse features. A neoadjuvant RT/CRT for cervical cancer is not routinely used outside of clinical trials. Here, according to local practice at our gynecological cancer center, the indication could be set after particularly intense discussion in the tumor board. Patients were informed in detail about the individual treatment character before informed consent was given.

### Radiation therapy and chemotherapy

**Fig. 1:**
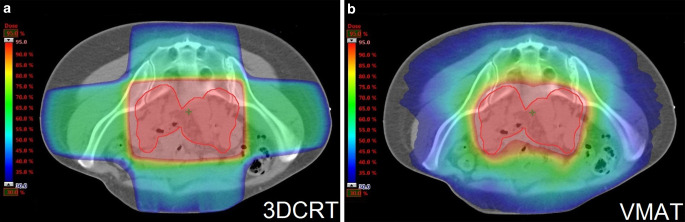
**a,** **b** Intraindividual comparison (transverse views) of dose distributions with a 3D conformal radiotherapy (*3DCRT*) plan (**a**) and a volumetric modulated arc therapy (*VMAT/RapidArc*®, Varian Medical Systems, Palo Alto, USA) plan (**b**). The color wash ranges from 95% to 30% of the prescribed dose of 50.4 Gy, the *thick red line *indicates the planning target volume

EBRT was applied with 6‑MeV or 20-MeV linear accelerator photons. The target volumes were defined according to the respective guidelines [21, 22]. The planning target volume was defined by adding a 10-mm margin to the clinical target volume. The International Commission on Radiation Units and Measurements (ICRU) reports provided the basis for plan calculation [23, 24]. In 3DCRT, a four-field box technique (anteroposterior/right and left lateral) was used. In definitive RT/CRT, a two-field technique (anteroposterior/posteroanterior) with central shielding was used for boost therapy [25]. VMAT was performed using RapidArc® (Varian Medical Systems, Palo Alto, USA). The treatment plans were calculated with the progressive resolution algorithm in Eclipse. These dose constraints were used for both 3DCRT and VMAT: small bowel ≥50 Gy in ≤10 cm^3^ volume and ≥40 Gy in ≤100 cm^3^ volume; rectum ≥65 Gy in ≤17% volume and ≥40 Gy in ≤50% volume; bladder ≥65 Gy in ≤25% volume and ≥40 Gy in ≤50% volume. Fig. 1a, b and Supplementary Figs. 1a, b and 2 illustrate a comparison of dose distributions and dose–volume histograms with 3DCRT and VMAT.

Where indicated, high-dose-rate brachytherapy was administered. In definitive RT/CRT, in patients with stages IB2–IVA, MRI was performed after EBRT. The treating radiation oncologist chose between an intracavitary or a combined intracavitary/interstitial approach, depending on tumor shrinkage and patient anatomy. The brachytherapy was delivered to a total dose of 24 Gy (weekly sessions of 6 Gy). In postoperative RT/CRT, in patients with close or positive vaginal margins, intracavitary brachytherapy was applied (10 Gy, two sessions of 5 Gy in 1 week).

Where indicated, chemotherapy was given concurrently with RT. Standardly, weekly cisplatin (40 mg/m^2^ total body surface area, total 240 mg/m^2^, six cycles) was administered. In cases of decreased renal function, a different regimen was selected or chemotherapy was omitted.

### Assessment of toxicity and follow-up

The Common Terminology Criteria for Adverse Events (CTCAE) criteria (version 5.0) [26] were used to assess acute toxicities. Patients were monitored at least weekly, including physical examination and the acquisition of blood samples. After RT/CRT, patients were monitored at least every second week until symptoms were satisfactorily controlled. The highest score of skin toxicity, proctitis, enteritis, and urinary toxicity was used to classify the grade of overall acute toxicity. The “Late Effects of Normal Tissues-subjective, objective, management, and analytic” (LENT-SOMA) criteria [27] were used to assess late toxicities. Patients were monitored at least annually for 5 years. The highest score of skin toxicity, proctitis, small bowel toxicity, urinary toxicity and vaginal toxicity was used to classify the grade of overall late toxicity.

### Statistics

The chi-square test (dichotomous variables), the Kendall’s tau test (ordinal variables), and the Mann–Whitney U test (continuous variables) were used for univariable comparison of patient characteristics and toxicity (cut-off *p* < 0.05). A multivariable model (ordinal logistic regression [28], cut-off *p* < 0.05) was established in cases of differences in toxicity endpoints in the univariable analysis. First, the variables were dichotomized (see Supplementary Table S1). Secondly, parameters with a tendency towards an influence on toxicity (*p* < 0.2) were included in the multivariable model. The survival times (overall survival, OS; progression-free survival, PFS; and locoregional control, LC) were calculated from the day of RT/CRT initiation. The log-rank test was performed to compare treatment groups (cut-off *p* < 0.05). We used SPSS v12.0 (IBM) for Kendall’s tau test, Mann–Whitney U test, and ordinal logistic regression. The chi-square test and the log-rank test were performed using STATISTICA v.10.0.1011.0 (StatSoft Inc.).

## Results

### Patients

In total, 105 consecutive patients (treatment between 11/1995 and 06/2014) met the inclusion criteria. Among these, 75 (71%) were treated with 3DCRT and 30 (29%) with VMAT. During the time period, 8 patients were irradiated with IMRT. Since this study focused on patients treated with VMAT, these patients were not considered in further analysis. Additionally, during the period, in 9 patients, the paraaortic region was included in treatment volumes. Due to the relevant bias for outcomes and toxicities, these patients were excluded from further analysis, too. In the study cohort, the median follow-up was 56.1 months (range 5.0–287.2) for the 3DCRT cohort and 29.3 months (range 5.2–65.3) for the VMAT cohort. Treatment groups were balanced in baseline clinical characteristics (Table 1).

**Table 1:** Patient characteristics

Parameter	Study group		*p*-value
	3D conformal radiotherapy	Volumetric modulated arc therapy	
**Age, years**^**b**^	55.2 (25–88)	56.3 (32–87)	0.9
**Body mass index**^**b**^	25.8 (15.7–45.9)	26.7 (19.8–40.5)	0.5
**FIGO stage**^**a**^			0.1
*I*	22 (29.4)	7 (23.3)	
*II*	25 (33.3)	12 (40.0)	
*III*	25 (33.3)	5 (16.7)	
*IV*	3 (4.0)	6 (20.0)	
**Histological subtype**^**a**^			0.8
*Squamous cell*	62 (82.7)	26 (86.7)	
*Non-squamous cell*	13 (17.3)	4 (13.3)	
Adenocarcinoma	11 (14.7)	3 (10.0)	
Adenosquamous	1 (1.3)	1 (3.3)	
Undifferentiated	1 (1.3)	0 (0.0)	
**Histologic grading**^**a, c**^			0.7
G1	2 (2.7)	1 (3.9)	
G2	56 (75.7)	18 (69.2)	
G3	16 (21.6)	7 (26.9)	

*FIGO* Fédération Internationale de Gynécologie et d`Obstétrique

^a^Data give the number of patients; the numbers in parentheses denote the percentage

^b^Data give the mean, the numbers in parentheses give the range

^c^The information on histologic grading is missing in five patients

### Radiation therapy and chemotherapy

Definitive RT/CRT was performed in 53 patients (50%), adjuvant RT/CRT was performed in 31 patients (30%), and neoadjuvant RT/CRT was applied in 21 patients (20%; Table 2). The reasons for omission of brachytherapy were patient refusal (*n* = 4), technical infeasibility (*n* = 6), and deterioration of patient condition (*n* = 1). In total, in 36/53 (68%) patients with definitive RT/RCT, in 23/31 patients (74%) with adjuvant RT/RCT, and in 21/21 patients (100%) with neoadjuvant RT/RCT, concomitant chemotherapy was applied. Patients who were not suitable for cisplatin received mitomycin C (*n* = 4), 5‑fluorouracil/mitomycin C (*n* = 1), or carboplatin (*n* = 1).

**Table 2:** Treatment characteristics

Parameter	Study group	
	3D conformal radiotherapy	Volumetric modulated arc therapy
**Treatment regimen**		
*Definitive*^*a*^	39 (52.0)	14 (46.7)
Brachytherapy^a^	32 (82.1)	10 (71.4)
Radiotherapy, total dose [Gy]^b^	70.1 (59.4–84.4)	69.7 (59.0–78.4)
Received planned dose	39 (100.0)	14 (100.0)
*Postoperative*^*a*^	25 (33.3)	6 (20.0)
Brachytherapy	4 (16.0)	0 (0.0)
Radiotherapy, total dose [Gy]^b^	51.1 (48.6–60.4)	50.4 (all patients)
Received planned dose	24 (96.0)	6 (100.0)
*Preoperative*^*a*^	11 (14.7)	10 (33.3)
Radiotherapy, total dose [Gy]^b^	46.0 (45.0–50.4)	45.5 (45.0–50.4)
Received planned dose	11 (100.0)	10 (100.0)
**Chemotherapy**^**a**^		
*Yes*	56 (74.7)	24 (80.0)
Received full dose	45 (80.4)	23 (95.8)
Received cisplatin	55 (98.2)	19 (79.2)

^a^Data give the number of patients; the numbers in parentheses denote the percentage

^b^Data give the mean, the numbers in parentheses give the range

### Outcome

There were no significant differences in outcome between 3DCRT-treated and VMAT-treated patients.

In patients who underwent definitive RT/CRT, the 2‑year OS was 61% for both 3DCRT and VMAT (*p* = 0.9). The 2‑year PFS was 80% for 3DCRT and 74% for VMAT (*p* = 0.5). The 2‑year LC was 85% for 3DCRT and 74% for VMAT (*p* = 0.6).

In patients who received adjuvant RT/CRT, 2‑year OS was 96% for 3DCRT and 100% for VMAT (*p* = 0.6). The 2‑year PFS was 88% for 3DCRT and 100% for VMAT (*p* = 0.5). The 2‑year LC was 96% for 3DCRT and 100% for VMAT (*p* = 0.6).

In patients who underwent neoadjuvant RT/CRT, the 2‑year OS was 82% for 3DCRT and 90% for VMAT (*p* = 0.7). The 2‑year PFS was 100% for 3DCRT and 86% for VMAT (*p* = 0.4). The 2‑year LC was 100% for both 3DCRT and VMAT (*p* = 0.3).

### Toxicity

Overall acute urinary toxicity occurred more frequently during VMAT treatment, whereas high-grade (≥grade 3) urinary toxicity occurred in only a very small number of patients (*n* = 1 for 3DCRT and VMAT, Table 3 [acute organ toxicity] and Supplementary Table S2 [hematologic toxicity]). The late toxicity data were available for 64 3DCRT-treated patients (85.3%) and for 26 VMAT-treated patients (86.7%). Late small bowel toxicity and overall late toxicity were significantly less frequent in the VMAT group (Table 4).

**Table 3:** Acute toxicity

Toxicity grade	Study group		*p*-value
	3D conformal radiotherapy	Volumetric modulated arc therapy	
*Skin*^*a*^			0.6^b^
0	15 (20.0)	4 (13.4)	
1	42 (56.0)	19 (63.3)	
2	17 (22.7)	6 (20.0)	
3	1 (1.3)	1 (3.3)	
*Proctitis*^*a*^			0.08^b^
0	22 (29.4)	3 (10.0)	
1	25 (33.3)	13 (43.3)	
2	27 (36.0)	13 (43.3)	
3	1 (1.3)	1 (3.4)	
*Enteritis*^*a*^			0.6^b^
0	30 (40.0)	12 (40.0)	
1	19 (25.4)	5 (16.7)	
2	25 (33.3)	12 (40.0)	
3	1 (1.3)	1 (3.3)	
*Urinary toxicity*^*a*^			0.03^b^*
0	45 (60.0)	11 (36.7)	
1	24 (32.0)	14 (46.7)	
2	5 (6.7)	4 (13.3)	
3	1 (1.3)	1 (3.3)	
*Overall acute toxicity*^*a*^			0.18^b^
0	1 (1.3)	0 (0.0)	
1	27 (36.0)	8 (26.7)	
2	44 (58.7)	19 (63.3)	
3	3 (4.0)	3 (10.0)	

*Statistically significant *p*-value

^a^Data give the number of patients; the numbers in parentheses denote the percentage

^b^Univariate comparison, Kendall’s tau test

**Table 4:** Late toxicity

Toxicity grade	Study group		*p‑value*
	3D conformal radiotherapy	Volumetric modulated arc therapy	
*Skin*^*a*^			0.9^b^
0	55 (85.9)	22 (84.6)	
1	8 (12.5)	4 (15.4)	
2	1 (1.6)	0 (0.0)	
*Proctitis*^*a*^			0.5^b^
0	42 (65.6)	20 (77.0)	
1	6 (9.4)	1 (3.8)	
2	8 (12.5)	1 (3.8)	
3	7 (10.9)	1 (3.8)	
4	1 (1.6)	3 (11.6)	
*Small bowel toxicity*^*a*^			<0.001^b^*
0	45 (70.2)	25 (96.2)	
1	4 (6.3)	0 (0.0)	
2	7 (10.9)	0 (0.0)	
3	4 (6.3)	1 (3.8)	
4	4 (6.3)	0 (0.0)	
*Urinary toxicity*^*a*^			0.1^b^
0	30 (46.9)	18 (69.2)	
1	17 (26.6)	3 (11.5)	
2	8 (12.5)	3 (11.5)	
3	7 (10.9)	0 (0.0)	
4	2 (3.1)	2 (7.8)	
*Overall late toxicity*^*a*^			0.04^b^*
0	19 (29.7)	15 (57.7)	
1	15 (23.4)	4 (15.5)	
2	12 (18.8)	3 (11.5)	
3	12 (18.8)	1 (3.8)	
4	6 (9.3)	3 (11.5)	

*Statistically significant *p*-value

^a^Data give the number of patients; the numbers in parentheses denote the percentage

^b^Univariate comparison, Kendall’s tau test

In multivariable analysis, the VMAT treatment was independently associated with an increased risk of acute urinary toxicity (*p* = 0.01, Table 5 and Supplementary Table S1). In VMAT-treated patients, the risk for late small bowel toxicity was significantly reduced (*p* = 0.03). The overall occurrence of late toxicity was significantly more frequent in patients with low BMI (*p* = 0.03) and in patients with overall acute toxicity ≥grade 2 (*p* < 0.01).

**Table 5:** Influence of radiotherapy technique and patient- and treatment-related parameters on toxicity

Parameter	Acute toxicity		Late toxicity			
	Urinary toxicity		Small bowel toxicity		Overall late toxicity	
	OR (CI)	*p-*value	OR (CI)^a^	*p-*value	OR (CI)	*p-*value
*Radiotherapy technique*^*a*^		*0.01*		*0.03*		0.1
3DCRT (75)	1.00	–	1.00	–	1.00	–
VMAT (30)	2.94 (1.27–6.67)	–	0.10 (0.01–0.78)	–	0.46 (0.18–1.16)	–
*Radiotherapy, total dose*^*a*^		0.4				
>50.4 Gy (65)	1.00	–	–	–	–	–
≤50.4 Gy (50)	0.58 (0.17–1.93)	–	–	–	–	–
*Brachytherapy*^*a*^		0.5				
Yes (46)	1.00	–	–	–	–	–
No (59)	0.67 (0.20–2.25)	–	–	–	–	–
*Hysterectomy prior to treatment*^*a*^				0.2		
Yes (31)	–	–	1.00	–	–	–
No (74)	–	–	0.52 (0.18–1.51)	–	–	–
*Acute toxicity, enteritis*^*a*^				0.1		
<grade 2 (66)	–	–	1.00	–	–	–
≥grade 2 (39)	–	–	2.56 (0.89–7.69)	–	–	–
*Body mass index*^*a,b*^						0.03*
Median: 25.4						
≥median (49)	–	–	–	–	1.00	–
<median (49)	–	–	–	–	2.46 (1.09–5.55)	–
*Overall acute toxicity*^*a*^						<0.01*
<grade 2 (36)	–	–	–	–	1.00	–
≥grade 2 (69)	–	–	–	–	4.17 (1.69–10.04)	–

*OR* odds ratio, *CI* confidence interval, *3DCRT* 3D conformal radiotherapy, *VMAT* volumetric modulated arc therapy

*Statistically significant *p*-value

^a^Parameters were preselected in univariate analysis for multivariate model, see Supplementary Table S1

^b^The information on body mass index is missing in seven patients

## Discussion

Within a prospective randomized trial, when comparing IMRT with 3DCRT, Gandhi et al. reported a comparable clinical outcome and a significant reduction of acute and chronic toxicity with IMRT [10]. Thus, high-quality evidence supports the wide use of IMRT in cervical cancer. Further studies compared planning results with VMAT to results with IMRT [13, 14, 29]. There appears a certain amount of heterogeneity in the results: Cozzi et al. and Sharfo et al. found similar target volume coverage, while Renard-Oldrini et al. found an improvement with VMAT [13, 14, 29]. While Cozzi et al. found improved organs at risk sparing, Sharfo et al. do not support this finding [13, 14]. However, in cervical cancer treatment, VMAT is used only in 26% of the radiation oncology facilities in Germany [30]. Due to the rareness of the disease, only a limited number of patients are treated per facility [30]. These aspects might explain that to date, only a few, mostly small studies have reported clinical results with VMAT [15–18]. A systematic comparison of VMAT with other EBRT techniques has only been occasionally reported [18]. We herein compared clinical results with VMAT to clinical results with conventional 3DCRT.

In our study, VMAT significantly reduced late small bowel toxicity. Late small bowel toxicity is known to be correlated with the bowel volume receiving higher radiation doses (≥50 Gy) [31]. Cozzi et al. demonstrated a great reduction of the bowel volume receiving ≥40 Gy with VMAT in cervical cancer. This reflects the technique’s potential to achieve a minimization of the high-dose volumes [13]. Our study indicates that these dosimetric advantages translate into clinical benefits. In the VMAT group, small bowel toxicity only occurred in 1 patient (3.8%). Due to the reduction of small bowel toxicity, an improvement in long-term morbidity is absolutely conceivable.

Additionally, it has to be considered that lesser side effects could result in a reduction of treatment breaks, and, consequently, in more effective local and systemic treatment. In our study, there were no differences in survival rates at 2 years. Similarly, previous studies have reported comparable survival rates with IMRT and conventional EBRT techniques [9, 11]. In a study by Roszak et al., gastrointestinal toxicity was the main reason for interruptions of RT/CRT [32], whereas the overall rates of severe gastrointestinal toxicity (≥grade 3) are lower than 10% for conventional and novel EBRT techniques [11, 16]. Similarly, we found ≥grade 3 overall acute toxicity in ≤10% of patients. However, of course, novel EBRT techniques should aim at reducing both severe and less pronounced side effects. Eventually, the already low rates of severe treatment-related toxicity with conventional EBRT techniques might leave limited space to attain improved outcome through a possible reduction of treatment breaks.

Interestingly, we found that the VMAT treatment was associated with an increased risk of acute urinary toxicity. During RT/CRT, genitourinary toxicity is less common than gastrointestinal toxicity, with relevant toxicities in only 1.5% of patients [4]. These low rates are comparable to ≥grade 3 urinary toxicity with VMAT in our study (3.3%), with VMAT in the study by Vandecaastele et al. (0%), and with IMRT in the study by Gandhi et al. (0%) [10, 16]. Gandhi et al. found no differences in rates of genitourinary toxicity rates when comparing IMRT and conventional 3DCRT [10]. The authors discuss that in their study’s 3DCRT-treated patients, the lack of blocks used could have led to higher genitourinary toxicity rates (here, ≥grade 3 toxicity in 13.6% of the patients) as compared to previous studies [10]. In our study, blocks were used for boost therapy in 3DCRT [25]. Thus, possibly due to the increase in the total volume of the bladder wall being exposed to irradiation with VMAT, higher toxicity rates might be explained. However, the increase was seen primarily in the <grade 3 toxicities. In line with other studies, severe acute urinary toxicity occurred in less than 5% of all patients [16, 17]. Thus, the significance for the whole patient population remains limited and increased attention should be paid to long-term side effects and quality of life, which are especially important from a patient perspective [5]. Finally, due to the relatively small sample size, the heterogeneity of the cohort, and the rare occurrence of genitourinary toxicity, an overinterpretation of the findings should be avoided.

In our study, a low BMI and acute toxicity ≥grade 2 were associated with increased overall late toxicity. Previous studies have demonstrated an influence of patient- or treatment-related parameters on side effects in RT/CRT of pelvic malignancies [32–37]. Furthermore, there is evidence that the severity of acute toxicity is correlated with the occurrence of late toxicity [36, 38]. First, we found that a low BMI was associated with a twofold-increased risk of overall late toxicity. In patients treated with CRT, the influence of bodily constitution on chemotherapy pharmacokinetics might explain the differences in damage to normal tissues [35]. Furthermore, the links between adipose tissue, chronic inflammation, and the immune system may provide a possible explanation [34]. Secondly, in our study, overall acute toxicity ≥grade 2 was associated with a fourfold-increased risk of overall late toxicity. This finding is in line with previous studies which found an association of acute toxicity and late toxicity in treatment of gynecologic malignancies [36, 38]. The predictive value of the BMI and of the occurrence of acute toxicity ≥grade 2 bear important implications for clinical practice. In both patient groups, close monitoring during follow-up is reasonable.

A retrospective single-center study may suffer from biases which could have distorted the results. Furthermore, we included patients with different treatment schedules, different radiation doses, different chemotherapy regimens or no concomitant chemotherapy administered, and different staging procedures (e.g., a relevant proportion of patients without surgical lymph node staging). Additionally, we did not include an analysis of dose–volume histograms in our study, which could further clarify the relationships between RT technique and side effects. The multivariable analysis, including patient- and treatment-related parameters, addressed these issues in part. Additionally, the long period of the study might have led to changes in local treatment practice. Since physician-dependent differences in the delineation of target volumes significantly contribute to heterogeneity in RT/CRT of cervical cancer [39], as previously reported, we developed strategies to improve treatment homogeneity [39]. The incidence of cervical cancer is low, and studies on VMAT treatment are rare. Thus, our study significantly contributes to the understanding of the role of VMAT and patient- and treatment-related parameters in RT/CRT of cervical cancer.

## Conclusion

We compared VMAT and 3DCRT for cervical cancer. We demonstrated reduced late small bowel toxicity with VMAT. An improvement in long-term morbidity is absolutely conceivable. VMAT-treated patients experienced acute urinary toxicity more frequently. Overall, the rates of high-grade urinary toxicity were very low, limiting the relevance of this finding. During follow-up, the close monitoring of patients with a low BMI and of patients who experienced acute toxicity ≥grade 2 could improve late toxicity profiles. Finally, modern irradiation techniques with lower rates of toxicity could pave the way for more effective systemic treatment options. This could result in a relevant improvement of outcomes and quality of life.

## Supplementary Information

Suppl. Fig. 1a. Intraindividual comparison (sagittal views) of dose distributions with a 3D conformal radiotherapy (3DCRT) plan (Suppl. Fig. 1a) and a volumetric modulated arc therapy (VMAT) plan (Suppl. Fig. 1b). The color wash ranges from 95 to 30% of the prescribed dose of 50.4 Gy, the *thick red line* indicates the planning target volume

Suppl. Fig. 1b. Intraindividual comparison (sagittal views) of dose distributions with a 3D conformal radiotherapy (3DCRT) plan (Suppl. Fig. 1a) and a volumetric modulated arc therapy (VMAT) plan (Suppl. Fig. 1b). The color wash ranges from 95 to 30% of the prescribed dose of 50.4 Gy, the *thick red line* indicates the planning target volume

Suppl. Fig. 2. Intraindividual comparison of dose–volume histograms (DVHs) with a 3D conformal radiotherapy (3DCRT) plan and a volumetric modulated arc therapy (VMAT) plan. The DVHs are illustrated for the planning target volume (PTV) and for the small bowel. The small bowel volume receiving higher radiation doses, which is of particular significance for the development of late complications, is reduced with VMAT

Supplementary Table S1. Preselection of patient- and treatment-related parameters for multivariate analysis

Supplementary Table S2. Hematologic toxicity
