# Supplementary figures and images for: Conventional 3D conformal radiotherapy and volumetric modulated arc therapy for cervical cancer: Comparison of clinical results with special consideration of the influence of patient- and treatment-related parameters
Source: Strahlenther Onkol. 2021 May 3;197(6):520–7. doi: 10.1007/s00066-021-01782-5 (PMC8154751; doi:10.1007/s00066-021-01782-5)

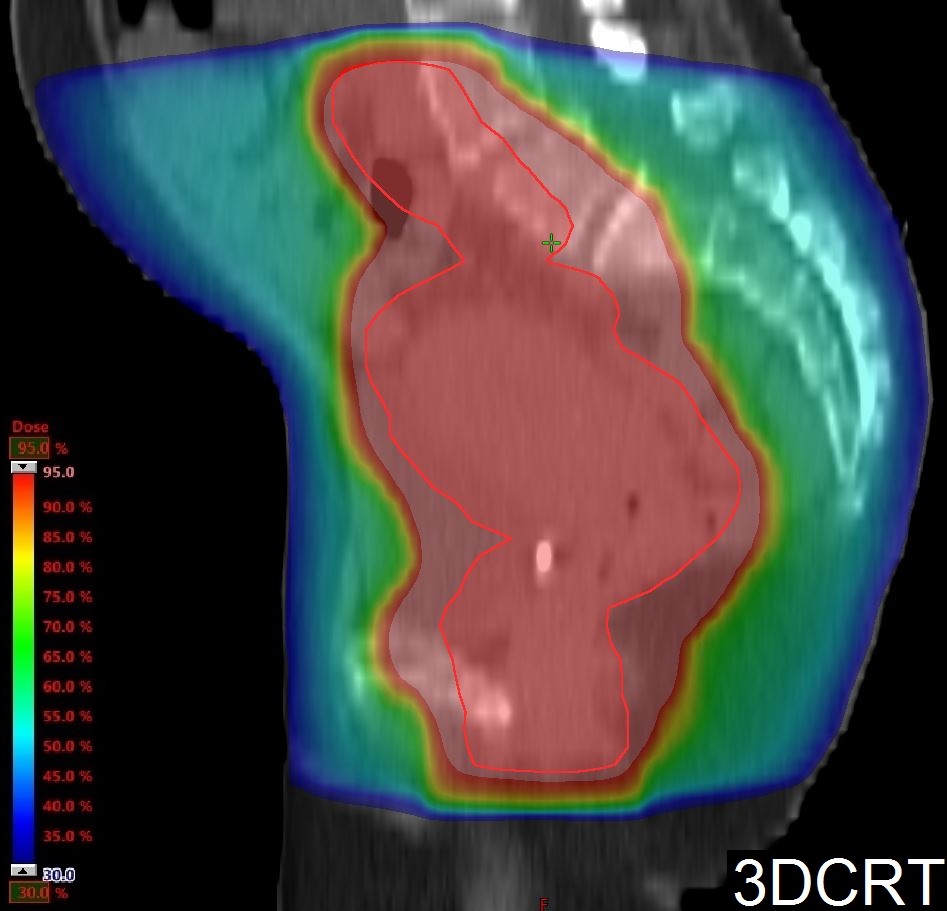

Supplement: Supplementary file 1 — Suppl. Fig. 1a. Intraindividual comparison (sagittal views) of dose distributions with a 3D conformal radiotherapy (3DCRT) plan (Suppl. Fig. 1a) and a volumetric modulated arc therapy (VMAT) plan (Suppl. Fig. 1b). The color wash ranges from 95 to 30% of the prescribed dose of 50.4 Gy, the thick red line indicates the planning target volume [file 66_2021_1782_MOESM1_ESM.jpg]

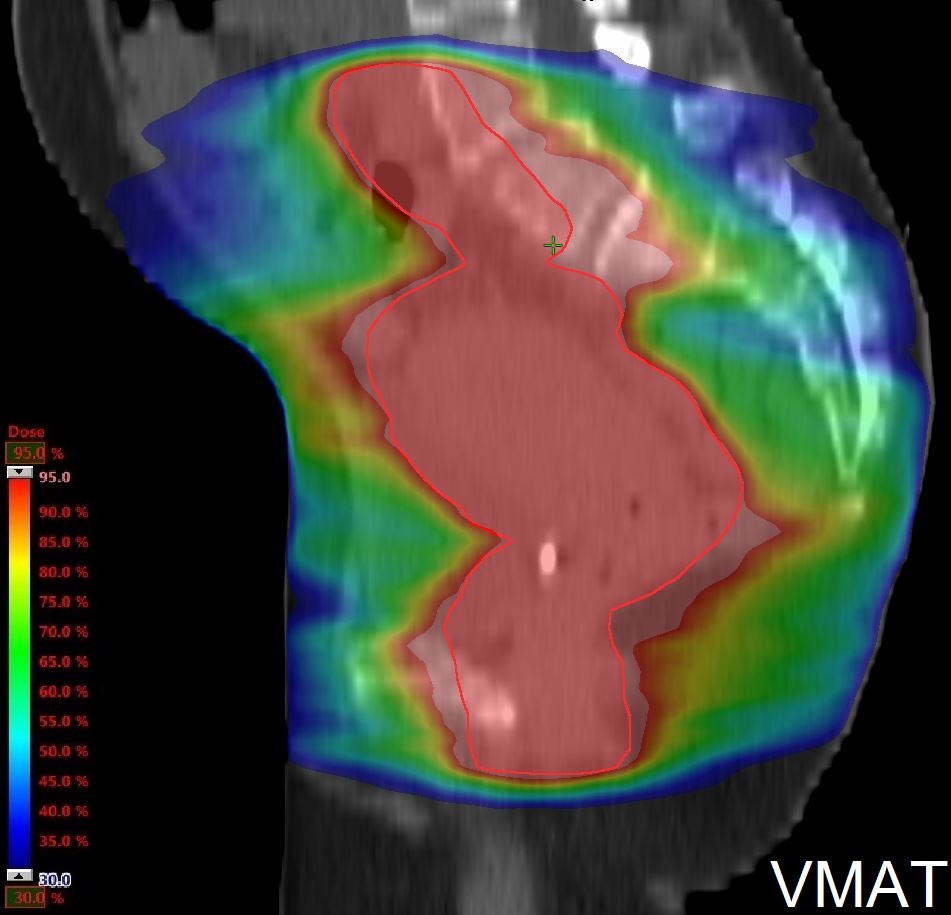

Supplement: Supplementary file 2 — Suppl. Fig. 1b. Intraindividual comparison (sagittal views) of dose distributions with a 3D conformal radiotherapy (3DCRT) plan (Suppl. Fig. 1a) and a volumetric modulated arc therapy (VMAT) plan (Suppl. Fig. 1b). The color wash ranges from 95 to 30% of the prescribed dose of 50.4 Gy, the thick red line indicates the planning target volume [file 66_2021_1782_MOESM2_ESM.jpg]

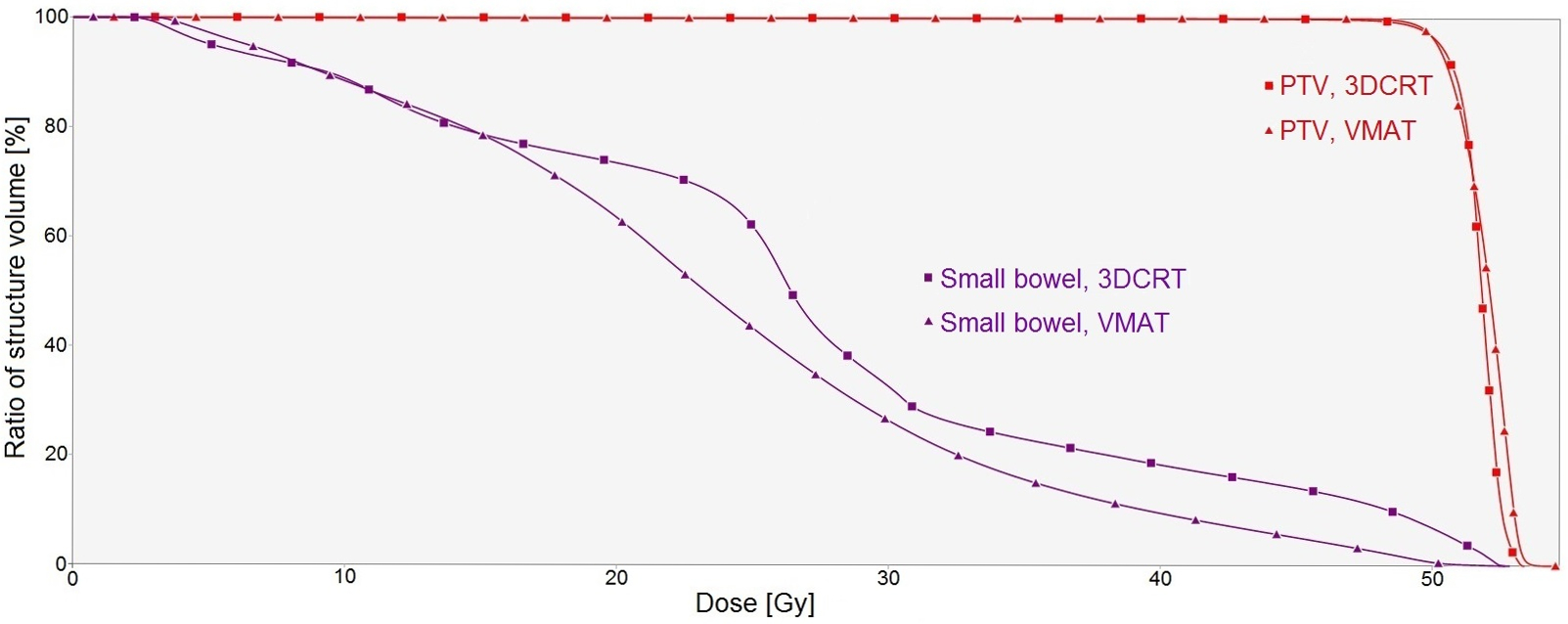

Supplement: Supplementary file 3 — Suppl. Fig. 2. Intraindividual comparison of dose–volume histograms (DVHs) with a 3D conformal radiotherapy (3DCRT) plan and a volumetric modulated arc therapy (VMAT) plan. The DVHs are illustrated for the planning target volume (PTV) and for the small bowel. The small bowel volume receiving higher radiation doses, which is of particular significance for the development of late complications, is reduced with VMAT [file 66_2021_1782_MOESM3_ESM.jpg]
